# *N*′-(4-Hydr­oxy-3-methoxy­benzyl­idene)-4-methoxy­benzohydrazide monohydrate

**DOI:** 10.1107/S1600536809034242

**Published:** 2009-08-29

**Authors:** Jiu-Fu Lu, Suo-Tian Min, Hong-Guang Ge, Xiao-Hui Ji

**Affiliations:** a School of Chemistry and Environmental Science, Shaanxi University of Technology, Hanzhong 723000, People’s Republic of China

## Abstract

In the title compound, C_16_H_16_N_2_O_4_·H_2_O, the dihedral angle between the two aromatic rings is 19.6 (2)°. In the crystal structure, mol­ecules are linked into a three-dimensional network by inter­molecular N—H⋯O, O—H⋯N and O—H⋯O hydrogen bonds.

## Related literature

For our previous work in this area, see: Lu *et al.* (2008*a* ▶,*b* ▶,*c* ▶). For related structures, see: Abdul Alhadi *et al.* (2009 ▶); Mohd Lair *et al.* (2009 ▶); Narayana *et al.* (2007 ▶). For reference bond-length data, see: Allen *et al.* (1987 ▶).
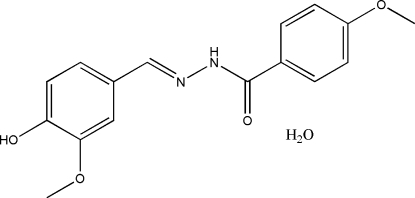


## Experimental

### 

#### Crystal data

C_16_H_16_N_2_O_4_·H_2_O
*M* *_r_* = 318.32
Monoclinic, 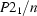

*a* = 7.942 (1) Å
*b* = 21.273 (2) Å
*c* = 10.246 (1) Å
β = 106.596 (2)°
*V* = 1659.0 (3) Å^3^
*Z* = 4
Mo *K*α radiation
μ = 0.10 mm^−1^
*T* = 298 K
0.32 × 0.30 × 0.30 mm

#### Data collection

Bruker APEXII CCD diffractometer
Absorption correction: multi-scan (*SADABS*; Sheldrick, 2004 ▶) *T*_min_ = 0.970, *T*_max_ = 0.972
9533 measured reflections
3338 independent reflections
2291 reflections with *I* > 2σ(*I*)
*R*_int_ = 0.024

#### Refinement

*R*[*F* ^2^ > 2σ(*F* ^2^)] = 0.044
*wR*(*F* ^2^) = 0.127
*S* = 1.04
3338 reflections
220 parameters
4 restraints
H atoms treated by a mixture of independent and constrained refinement
Δρ_max_ = 0.20 e Å^−3^
Δρ_min_ = −0.18 e Å^−3^

### 

Data collection: *APEX2* (Bruker, 2004 ▶); cell refinement: *SAINT* (Bruker, 2004 ▶); data reduction: *SAINT*; program(s) used to solve structure: *SHELXS97* (Sheldrick, 2008 ▶); program(s) used to refine structure: *SHELXL97* (Sheldrick, 2008 ▶); molecular graphics: *SHELXTL* (Sheldrick, 2008 ▶); software used to prepare material for publication: *SHELXTL*.

## Supplementary Material

Crystal structure: contains datablocks global, I. DOI: 10.1107/S1600536809034242/hb5071sup1.cif

Structure factors: contains datablocks I. DOI: 10.1107/S1600536809034242/hb5071Isup2.hkl

Additional supplementary materials:  crystallographic information; 3D view; checkCIF report

## Figures and Tables

**Table 1:** Hydrogen-bond geometry (Å, °)

*D*—H⋯*A*	*D*—H	H⋯*A*	*D*⋯*A*	*D*—H⋯*A*
O5—H5*A*⋯N1^i^	0.846 (9)	2.487 (15)	3.1257 (18)	133.0 (17)
O5—H5*A*⋯O3^i^	0.846 (9)	2.107 (13)	2.8927 (18)	154 (2)
O5—H5*B*⋯O3^ii^	0.856 (9)	1.884 (10)	2.7401 (17)	179 (2)
N2—H2*B*⋯O2^i^	0.895 (9)	2.185 (12)	3.0398 (18)	159.6 (19)
O2—H2⋯O5^iii^	0.82	1.77	2.5775 (17)	170

Symmetry codes: (i) 
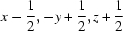
; (ii) 
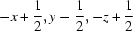
; (iii) 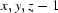.

**Fig. 1.**
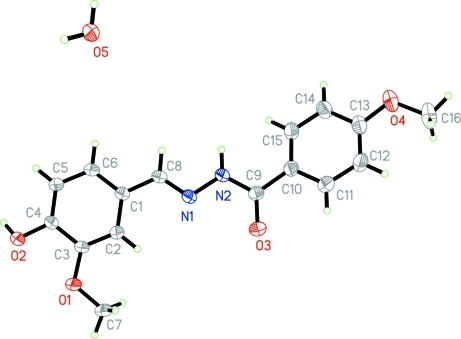
The molecular structure of (I) showing 30% probability displacement ellipsoids.

**Fig. 2.**
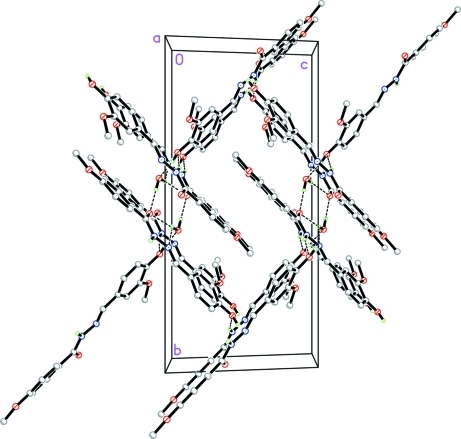
The crystal packing of (I) viewed along the a axis. Intermolecular hydrogen bonds are drawn by dashed lines. H atoms unrelated to the hydrogen bonding are omitted for clarity.

## supplementary crystallographic information

### Comment

Schiff bases and their metal complexes have received much attention in recent years. As part of our investigation on the crystal structures of Schiff bases derived from the condensation of aldehydes with benzohydrazides (Lu *et al.*,  2008*a*,b,c), we report herein the crystal structure of the title new Schiff base compound, (I).

The title compound (Fig. 1), consists of a Schiff base molecule and a water molecule of crystallization. The bond lengths have normal values (Allen *et al.*,  1987), and are comparable to those observed in similar compounds (Abdul Alhadi *et al.*,  2009; Mohd Lair *et al.*, 2009; Narayana *et al.*,  2007). The dihedral angle between the two aromatic rings is 19.6 (2)°, indicating that they the molecule is twisted.

In the crystal structure, the molecules are linked into a three-dimensional network by intermolecular N—H···O, O—H···N and O—H···O hydrogen bonds (Table 1 and Fig. 2).

### Experimental

The title compound was prepared by the Schiff base condensation of 4-hydroxy-3-methoxybenzaldehyde (0.1 mol) and 4-methoxybenzohydrazide (0.1 mmol) in 95% ethanol (50 ml). The excess ethanol was removed by distillation. The colourless solid obtained was filtered and washed with ethanol. Colourless blokcs of (I) were obatined by slow evaporation of a 95% ethanol solution at room temperature.

### Refinement

The imino H atom and water H atoms were located in a difference map and refined with N—H, O—H, and H···H distance restraint of 0.90 (1), 0.85 (1), and 1.37 (2) Å, respectively. Other H atoms were positioned geometrically (C—H = 0.93-0.97 Å, O—H = 0.82 Å) and refined using a riding model, with U_iso_(H) = 1.2U_eq_(C) and 1.5U_eq_(C_methyl_ and O).

### Crystal data

**Table:**

C_16_H_16_N_2_O_4_·H_2_O	*F*(000) = 672
*M**_r_* = 318.32	*D*_x_ = 1.274 Mg m^−^^3^
Monoclinic, *P*2_1_/*n*	Mo *K*α radiation, λ = 0.71073 Å
Hall symbol: -P 2yn	Cell parameters from 2203 reflections
*a* = 7.942 (1) Å	θ = 2.3–24.6°
*b* = 21.273 (2) Å	µ = 0.10 mm^−^^1^
*c* = 10.246 (1) Å	*T* = 298 K
β = 106.596 (2)°	Block, colourless
*V* = 1659.0 (3) Å^3^	0.32 × 0.30 × 0.30 mm
*Z* = 4	

### Data collection

**Table:**

Bruker APEXII CCD diffractometer	3338 independent reflections
Radiation source: fine-focus sealed tube	2291 reflections with *I* > 2σ(*I*)
graphite	*R*_int_ = 0.024
ω scans	θ_max_ = 26.3°, θ_min_ = 1.9°
Absorption correction: multi-scan (*SADABS*; Sheldrick, 2004)	*h* = −9→9
*T*_min_ = 0.970, *T*_max_ = 0.972	*k* = −26→25
9533 measured reflections	*l* = −12→11

### Refinement

**Table:**

Refinement on *F*^2^	Primary atom site location: structure-invariant direct methods
Least-squares matrix: full	Secondary atom site location: difference Fourier map
*R*[*F*^2^ > 2σ(*F*^2^)] = 0.044	Hydrogen site location: inferred from neighbouring sites
*wR*(*F*^2^) = 0.127	H atoms treated by a mixture of independent and constrained refinement
*S* = 1.04	*w* = 1/[σ^2^(*F*_o_^2^) + (0.0586*P*)^2^ + 0.2417*P*] where *P* = (*F*_o_^2^ + 2*F*_c_^2^)/3
3338 reflections	(Δ/σ)_max_ = 0.001
220 parameters	Δρ_max_ = 0.20 e Å^−^^3^
4 restraints	Δρ_min_ = −0.18 e Å^−^^3^

### Special details

**Table:**

Geometry. All esds (except the esd in the dihedral angle between two l.s. planes) are estimated using the full covariance matrix. The cell esds are taken into account individually in the estimation of esds in distances, angles and torsion angles; correlations between esds in cell parameters are only used when they are defined by crystal symmetry. An approximate (isotropic) treatment of cell esds is used for estimating esds involving l.s. planes.
Refinement. Refinement of F^2^ against ALL reflections. The weighted R-factor wR and goodness of fit S are based on F^2^, conventional R-factors R are based on F, with F set to zero for negative F^2^. The threshold expression of F^2^ > 2sigma(F^2^) is used only for calculating R-factors(gt) etc. and is not relevant to the choice of reflections for refinement. R-factors based on F^2^ are statistically about twice as large as those based on F, and R- factors based on ALL data will be even larger.

### Fractional atomic coordinates and isotropic or equivalent isotropic displacement parameters (Å^2^)

**Table:**

	*x*	*y*	*z*	*U*_iso_*/*U*_eq_	
O1	0.45414 (18)	0.25435 (6)	−0.34503 (15)	0.0726 (4)	
O2	0.25510 (15)	0.15740 (6)	−0.42933 (13)	0.0570 (3)	
H2	0.1742	0.1332	−0.4638	0.085*	
O3	0.32814 (18)	0.47213 (6)	0.11553 (15)	0.0682 (4)	
O4	−0.1311 (2)	0.60007 (7)	0.46039 (15)	0.0791 (4)	
O5	0.01680 (17)	0.08043 (6)	0.43784 (15)	0.0612 (4)	
N1	0.16872 (18)	0.37019 (6)	−0.01397 (14)	0.0489 (4)	
N2	0.10078 (19)	0.40488 (7)	0.07401 (15)	0.0508 (4)	
C1	0.1256 (2)	0.28010 (8)	−0.15935 (17)	0.0477 (4)	
C2	0.2726 (2)	0.29018 (8)	−0.20479 (17)	0.0487 (4)	
H2A	0.3435	0.3251	−0.1744	0.058*	
C3	0.3135 (2)	0.24897 (8)	−0.29416 (17)	0.0475 (4)	
C4	0.2067 (2)	0.19639 (8)	−0.34092 (17)	0.0456 (4)	
C5	0.0626 (2)	0.18616 (9)	−0.29573 (19)	0.0562 (5)	
H5	−0.0079	0.1511	−0.3256	0.067*	
C6	0.0218 (2)	0.22785 (9)	−0.2059 (2)	0.0600 (5)	
H6	−0.0768	0.2207	−0.1762	0.072*	
C7	0.5593 (3)	0.30919 (11)	−0.3109 (3)	0.0894 (8)	
H7A	0.4872	0.3458	−0.3389	0.134*	
H7B	0.6494	0.3084	−0.3565	0.134*	
H7C	0.6125	0.3105	−0.2142	0.134*	
C8	0.0764 (2)	0.32266 (8)	−0.06566 (18)	0.0512 (4)	
H8	−0.0267	0.3149	−0.0425	0.061*	
C9	0.1854 (2)	0.45679 (8)	0.13293 (18)	0.0507 (4)	
C10	0.1005 (2)	0.49380 (8)	0.21918 (18)	0.0510 (4)	
C11	0.1991 (3)	0.53788 (10)	0.3066 (2)	0.0729 (6)	
H11	0.3163	0.5435	0.3099	0.087*	
C12	0.1273 (3)	0.57418 (10)	0.3899 (2)	0.0757 (6)	
H12	0.1966	0.6033	0.4491	0.091*	
C13	−0.0457 (3)	0.56687 (9)	0.38450 (19)	0.0602 (5)	
C14	−0.1466 (3)	0.52298 (9)	0.2976 (2)	0.0623 (5)	
H14	−0.2638	0.5174	0.2945	0.075*	
C15	−0.0741 (3)	0.48746 (8)	0.21579 (19)	0.0568 (5)	
H15	−0.1440	0.4584	0.1566	0.068*	
C16	−0.0366 (4)	0.64796 (11)	0.5474 (2)	0.0959 (8)	
H16A	0.0642	0.6299	0.6119	0.144*	
H16B	−0.1111	0.6672	0.5951	0.144*	
H16C	0.0012	0.6791	0.4941	0.144*	
H2B	0.0004 (18)	0.3933 (10)	0.090 (2)	0.080*	
H5B	0.064 (2)	0.0464 (7)	0.421 (2)	0.080*	
H5A	−0.064 (2)	0.0719 (9)	0.473 (2)	0.080*	

### Atomic displacement parameters (Å^2^)

**Table:**

	*U*^11^	*U*^22^	*U*^33^	*U*^12^	*U*^13^	*U*^23^
O1	0.0662 (8)	0.0727 (9)	0.0998 (10)	−0.0254 (7)	0.0572 (8)	−0.0298 (8)
O2	0.0520 (7)	0.0565 (7)	0.0720 (8)	−0.0056 (6)	0.0329 (7)	−0.0161 (6)
O3	0.0697 (9)	0.0606 (8)	0.0880 (10)	−0.0194 (7)	0.0443 (8)	−0.0096 (7)
O4	0.1010 (11)	0.0712 (9)	0.0694 (9)	0.0105 (8)	0.0311 (8)	−0.0178 (8)
O5	0.0558 (8)	0.0502 (7)	0.0862 (10)	−0.0013 (6)	0.0342 (7)	−0.0118 (7)
N1	0.0522 (8)	0.0479 (8)	0.0549 (8)	−0.0001 (7)	0.0285 (7)	−0.0016 (7)
N2	0.0560 (9)	0.0487 (8)	0.0582 (9)	−0.0065 (7)	0.0329 (7)	−0.0072 (7)
C1	0.0480 (9)	0.0491 (9)	0.0524 (10)	−0.0014 (7)	0.0249 (8)	−0.0019 (8)
C2	0.0498 (10)	0.0460 (9)	0.0557 (10)	−0.0071 (8)	0.0241 (8)	−0.0036 (8)
C3	0.0423 (9)	0.0515 (9)	0.0561 (10)	−0.0040 (7)	0.0259 (8)	−0.0023 (8)
C4	0.0455 (9)	0.0455 (9)	0.0509 (10)	0.0010 (7)	0.0219 (8)	−0.0026 (7)
C5	0.0515 (10)	0.0550 (10)	0.0704 (12)	−0.0125 (8)	0.0307 (9)	−0.0129 (9)
C6	0.0529 (11)	0.0630 (11)	0.0772 (13)	−0.0131 (9)	0.0397 (10)	−0.0128 (10)
C7	0.0736 (14)	0.0922 (16)	0.125 (2)	−0.0380 (13)	0.0656 (15)	−0.0365 (15)
C8	0.0509 (10)	0.0531 (10)	0.0587 (11)	−0.0051 (8)	0.0304 (8)	−0.0027 (8)
C9	0.0588 (11)	0.0451 (9)	0.0536 (10)	−0.0068 (8)	0.0247 (8)	0.0040 (8)
C10	0.0649 (11)	0.0415 (9)	0.0511 (10)	−0.0042 (8)	0.0238 (9)	0.0016 (8)
C11	0.0790 (14)	0.0676 (13)	0.0817 (14)	−0.0244 (11)	0.0383 (12)	−0.0179 (11)
C12	0.0937 (17)	0.0644 (13)	0.0748 (14)	−0.0245 (12)	0.0335 (12)	−0.0234 (11)
C13	0.0831 (14)	0.0490 (10)	0.0522 (11)	0.0069 (10)	0.0252 (10)	−0.0008 (8)
C14	0.0600 (12)	0.0637 (12)	0.0634 (12)	0.0082 (9)	0.0179 (10)	−0.0075 (10)
C15	0.0593 (11)	0.0528 (10)	0.0574 (11)	0.0019 (8)	0.0151 (9)	−0.0089 (9)
C16	0.136 (2)	0.0756 (14)	0.0731 (15)	0.0114 (15)	0.0258 (15)	−0.0260 (13)

### Geometric parameters (Å, °)

**Table:**

O1—C3	1.3654 (19)	C5—H5	0.9300
O1—C7	1.419 (2)	C6—H6	0.9300
O2—C4	1.3622 (19)	C7—H7A	0.9600
O2—H2	0.8200	C7—H7B	0.9600
O3—C9	1.241 (2)	C7—H7C	0.9600
O4—C13	1.365 (2)	C8—H8	0.9300
O4—C16	1.419 (3)	C9—C10	1.482 (2)
O5—H5B	0.856 (9)	C10—C11	1.376 (3)
O5—H5A	0.846 (9)	C10—C15	1.384 (3)
N1—C8	1.272 (2)	C11—C12	1.388 (3)
N1—N2	1.3878 (18)	C11—H11	0.9300
N2—C9	1.343 (2)	C12—C13	1.369 (3)
N2—H2B	0.895 (9)	C12—H12	0.9300
C1—C6	1.385 (2)	C13—C14	1.378 (3)
C1—C2	1.391 (2)	C14—C15	1.372 (2)
C1—C8	1.452 (2)	C14—H14	0.9300
C2—C3	1.372 (2)	C15—H15	0.9300
C2—H2A	0.9300	C16—H16A	0.9600
C3—C4	1.403 (2)	C16—H16B	0.9600
C4—C5	1.369 (2)	C16—H16C	0.9600
C5—C6	1.382 (2)		
			
C3—O1—C7	117.57 (14)	H7B—C7—H7C	109.5
C4—O2—H2	109.5	N1—C8—C1	122.59 (15)
C13—O4—C16	117.99 (19)	N1—C8—H8	118.7
H5B—O5—H5A	109.9 (17)	C1—C8—H8	118.7
C8—N1—N2	114.08 (13)	O3—C9—N2	120.79 (16)
C9—N2—N1	119.46 (14)	O3—C9—C10	122.43 (15)
C9—N2—H2B	120.1 (14)	N2—C9—C10	116.78 (15)
N1—N2—H2B	120.5 (14)	C11—C10—C15	117.57 (17)
C6—C1—C2	118.84 (15)	C11—C10—C9	118.60 (17)
C6—C1—C8	118.80 (15)	C15—C10—C9	123.83 (16)
C2—C1—C8	122.36 (15)	C10—C11—C12	121.5 (2)
C3—C2—C1	120.41 (15)	C10—C11—H11	119.3
C3—C2—H2A	119.8	C12—C11—H11	119.3
C1—C2—H2A	119.8	C13—C12—C11	119.72 (19)
O1—C3—C2	125.22 (15)	C13—C12—H12	120.1
O1—C3—C4	114.67 (14)	C11—C12—H12	120.1
C2—C3—C4	120.11 (14)	O4—C13—C12	125.08 (18)
O2—C4—C5	123.37 (15)	O4—C13—C14	115.25 (19)
O2—C4—C3	117.03 (14)	C12—C13—C14	119.67 (18)
C5—C4—C3	119.59 (15)	C15—C14—C13	120.01 (19)
C4—C5—C6	120.06 (16)	C15—C14—H14	120.0
C4—C5—H5	120.0	C13—C14—H14	120.0
C6—C5—H5	120.0	C14—C15—C10	121.56 (17)
C5—C6—C1	120.98 (16)	C14—C15—H15	119.2
C5—C6—H6	119.5	C10—C15—H15	119.2
C1—C6—H6	119.5	O4—C16—H16A	109.5
O1—C7—H7A	109.5	O4—C16—H16B	109.5
O1—C7—H7B	109.5	H16A—C16—H16B	109.5
H7A—C7—H7B	109.5	O4—C16—H16C	109.5
O1—C7—H7C	109.5	H16A—C16—H16C	109.5
H7A—C7—H7C	109.5	H16B—C16—H16C	109.5

### Hydrogen-bond geometry (Å, °)

**Table:**

*D*—H···*A*	*D*—H	H···*A*	*D*···*A*	*D*—H···*A*
O5—H5A···N1^i^	0.85 (1)	2.49 (2)	3.1257 (18)	133 (2)
O5—H5A···O3^i^	0.85 (1)	2.11 (1)	2.8927 (18)	154 (2)
O5—H5B···O3^ii^	0.86 (1)	1.88 (1)	2.7401 (17)	179 (2)
N2—H2B···O2^i^	0.90 (1)	2.19 (1)	3.0398 (18)	160 (2)
O2—H2···O5^iii^	0.82	1.77	2.5775 (17)	170

Symmetry codes: (i) *x*−1/2, −*y*+1/2, *z*+1/2; (ii) −*x*+1/2, *y*−1/2, −*z*+1/2; (iii) *x*, *y*, *z*−1.
